# Deriving and validating a brief measure of treatment burden to assess person-centered healthcare quality in primary care: a multi-method study

**DOI:** 10.1186/s12875-020-01291-x

**Published:** 2020-10-28

**Authors:** David T. Eton, Mark Linzer, Deborah H. Boehm, Catherine E. Vanderboom, Elizabeth A. Rogers, Marlene H. Frost, Mike Wambua, Miamoua Vang, Sara Poplau, Minji K. Lee, Roger T. Anderson

**Affiliations:** 1 Department of Health Sciences Research, Mayo Clinic, Harwick Building, Second Floor, 200 First St SW, Rochester, MN 55905 USA; 2 Robert D. and Patricia E. Kern Center for the Science of Health Care Delivery, Mayo Clinic, Rochester, MN USA; 3 Hennepin Healthcare, Minneapolis, MN USA; 4 University of Minnesota Medical School, Minneapolis, MN USA; 5 Decision Partners for Health, Minneapolis, MN USA; 6 Women’s Cancer Program, Mayo Clinic, Rochester, MN USA; 7 Hennepin Healthcare Research Institute, Minneapolis, MN USA; 8 Department of Public Health Sciences, University of Virginia School of Medicine, Charlottesville, VA USA

**Keywords:** Quality of health care, Primary health care, Multimorbidity, Patient-reported outcome measures, Patient-reported experience measures, Quality of life, Patient-centered

## Abstract

**Background:**

In primary care there is a need for more quality measures of person-centered outcomes, especially ones applicable to patients with multiple chronic conditions (MCCs). The aim of this study was to derive and validate a short-form version of the Patient Experience with Treatment and Self-management (PETS), an established measure of treatment burden, to help fill the gap in quality measurement.

**Methods:**

Patient interviews (30) and provider surveys (30) were used to winnow items from the PETS (60 items) to a subset targeting person-centered care quality. Results were reviewed by a panel of healthcare providers and health-services researchers who finalized a pilot version. The Brief PETS was tested in surveys of 200 clinic and 200 community-dwelling MCC patients. Surveys containing the Brief PETS and additional measures (e.g., health status, medication adherence, quality of care, demographics) were administered at baseline and follow-up. Correlations and t-tests were used to assess validity, including responsiveness to change of the Brief PETS. Effect sizes (ES) were calculated on mean differences.

**Results:**

Winnowing and panel review resulted in a 34-item Brief PETS pilot measure that was tested in the combined sample of 400 (mean age = 57.9 years, 50% female, 48% white, median number of conditions = 5). Reliability of most scales was acceptable (alpha > 0.70). Brief PETS scores were associated with age, income, health status, and quality of chronic illness care at baseline (*P* < .05; rho magnitude range: 0.16–0.66). Furthermore, Brief PETS scores differentiated groups based on marital and education status, presence/absence of a self-management routine, and optimal/suboptimal medication adherence (*P* < .05; ES range: 0.25–1.00). Declines in patient-reported physical or mental health status over time were associated with worsening PETS burden scores, while improvements were associated with improving PETS burden scores (*P* < .05; ES range: 0.04–0.44). Among clinic patients, 91% were willing to complete the Brief PETS as part of their clinic visits.

**Conclusions:**

The Brief PETS (final version: 32 items) is a reliable and valid tool for assessing person-centered care quality related to treatment burden. It holds promise as a means of giving voice to patient concerns about the complexity of disease management.

## Background

Beyond their use in clinical research, patient-reported measures are increasingly being used for purposes of accountability, performance, and quality assessment of healthcare providers [1–4]. National and international initiatives have prioritized the standardization of survey and patient-reported outcome measures (PROMs) to help capture the experiences and outcomes that matter most to patients treated for chronic health conditions [5–7]. For instance, the International Consortium for Health Outcomes Measurement (ICHOM), an independent consortium of clinical experts, is working to identify core sets of standard outcome measures to use in monitoring patient outcomes with the goal of using such data to inform improvements in the quality and value of healthcare service delivery [4, 5]. To date, most of ICHOM’s published standard sets identify outcomes for individual conditions [8].

The standard approach to evaluating quality of care in people with multiple chronic conditions (MCCs) has relied on aggregating quality indicators for multiple single diseases (the “additive model”) [4], but there is little evidence that supports the validity of this approach [9]. In 2012, to address measurement gaps in MCCs, the National Quality Forum in the USA called for promotion of “cross-cutting measures” that can be applied across a variety of conditions and highlighted the need for further concentrated measure development, including assessment of patients’ experience with care and self-management [6]. A recent scoping review has shown that while some advances have been made, the need remains for good quality measures *specific to multi-morbid patients* or *non-specific, but robust in the presence of multi-morbidity* [4].

In addition to standard PROMs of health status and well-being, there has been considerable investment in measuring the patient’s experiences with healthcare services and providers through the use of patient-related experience measures or PREMs. A PREM assesses perceptions of patient-centered care by tapping aspects of the structure and processes of care from the perspective of the patient [10]. This can include perceptions of care delivery (e.g., patient satisfaction), experience with healthcare services and providers (e.g., patient-provider communication, coordination of care), and patient activation (e.g., shared decision-making). The Consumer Assessment of Healthcare Providers and Systems (CAHPS) program has developed and promotes assessments of consumers’ experiences with healthcare services and delivery by providers at the *point of clinical care* which is usually, but not always, a healthcare facility [11]. However, much of healthcare for chronic health conditions occurs outside of medical facilities and away from formal healthcare providers in the form of requisite self-management (i.e., tasks and activities that patients are asked to perform on their own in order to maintain optimal health). Currently, there is no quality measure available that addresses the ease with which healthcare-provider prescribed self-management is integrated into a patient’s daily life *outside* of formal healthcare settings, and which can be included as a basis for patient management and decision making. Yet this too may be an important marker of healthcare quality given the volume and complexity of provider prescribed self-care tasks especially for those with multi-morbidity [12].

We have studied self-care task complexity within the context of treatment burden. Treatment burden includes the workload of treatment and self-management for chronic health conditions, its impact on patient functioning, and stressors that exacerbate burden like financial concerns and difficulties with healthcare services [13, 14]. Treatment burden is especially relevant to people with MCCs who are often faced with the challenge of seamlessly integrating a complex self-care regimen into daily life [12, 15]. The ability to meet this challenge is important for both patients and healthcare providers. From the patient’s perspective, lower treatment burden is associated with better well-being and quality of life [16–19]. From the provider’s perspective, lower treatment burden in patients is associated with more adherence to prescribed medical regimens, including medication, diet, and exercise regimens [20–24]. Better adherence can lower the risk of disease exacerbations [25], and result in lower rates of hospitalization [25, 26], readmission [26, 27], and mortality [25, 26, 28]. Hence, measuring treatment burden could potentially inform healthcare providers of patient self-management challenges that if addressed might result in better patient outcomes. A provider’s ability to recognize treatment burden and attend to it accordingly could become a marker of good care quality.

We recently developed a comprehensive patient-reported measure of treatment burden – the Patient Experience with Treatment and Self-Management or PETS [14, 18]. The PETS assesses a range of generic treatment and self-management issues that cut across disease and treatment types. Its content was informed by patients with MCCs [13, 14], and it has demonstrated validity and responsiveness when used in this population [18, 29, 30]. The PETS conceptual framework [13] has also informed the conceptual foundations of other patient-reported measures of treatment burden, including ones developed in the UK [17] and France [31], and the PETS measure has been translated into other languages for use in select European populations [32]. The current version of the PETS (version 2.0) was developed for use in research. Its length (60 items) makes it less well-suited for regular use in practice settings. The aim of this study was to create a shorter version of the PETS, adapted from the longer form, and tailored specifically to the measurement of person-centered care quality. It is “person-centered” because the PETS assesses treatment and self-management challenges globally from the perspective of the person experiencing them without focusing on the specific illnesses or medical conditions being treated [33, 34].

## Overview of study methods

### Study design

**Fig. 1:**
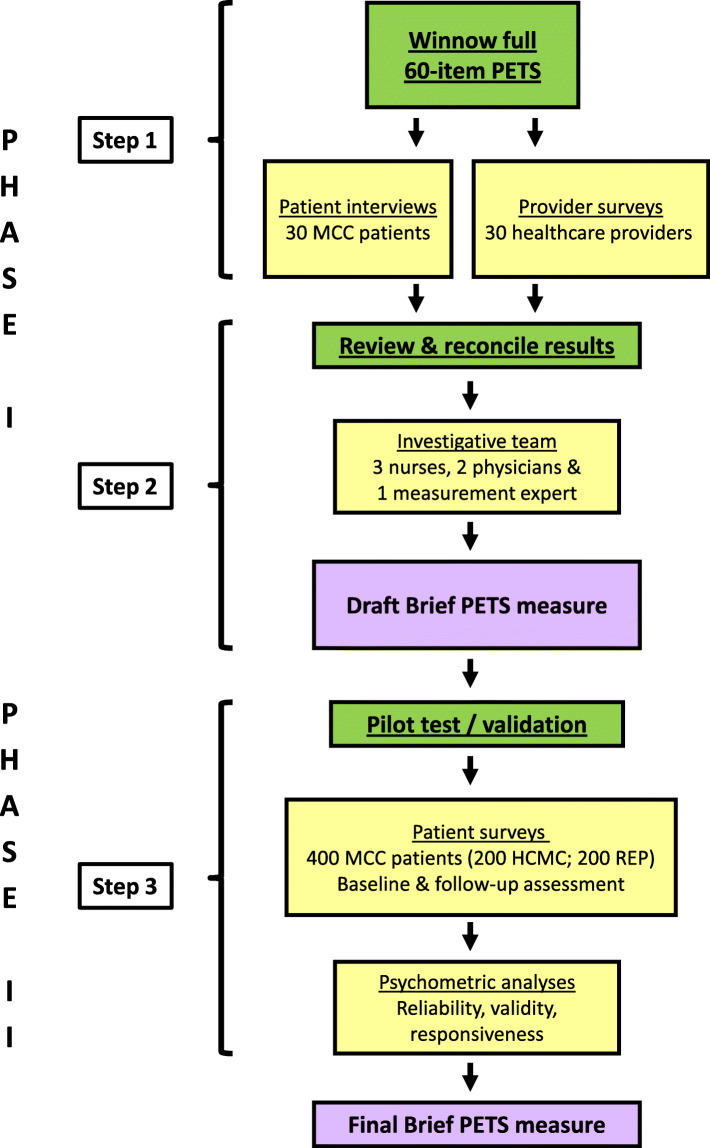
Study flow of work. Legend: PETS: Patient Experience with Treatment and Self-management; MCC: Multiple Chronic Conditions; HCMC: Hennepin County Medical Center; REP: Rochester Epidemiology Project

The project proceeded in two phases: derivation of a brief measure of treatment burden (Phase I) and pilot testing/validation (Phase II). The work flow is outlined in Fig. 1. In Phase I, a mixed-methods study design featuring patient interviews, healthcare provider surveys and review/input from key stakeholders was used to derive a pilot measure. The derivation phase emphasized creation of a measure with two particularly desirable attributes for a patient-reported quality measure: (a) short length to facilitate adoption and (b) content relevant to both patients and healthcare providers, and representative of issues that could be modified to improve care [1, 2]. The resulting measure was then tested in a prospective survey study of 400 outpatients with MCCs from two different healthcare systems and settings in the state of Minnesota, USA.

### Phase I - deriving the brief PETS-quality measure

A full description of the methods and results of the derivation phase (Steps 1 and 2 of Fig. 1) can be found in Additional Files 1 and 2, attached as supplements to this report. In brief, 30 MCC patients and 30 healthcare providers caring for MCC patients participated in exercises designed to winnow items from the full 60-item PETS measure into a subset considered appropriate for specification of treatment burden as a person-centered quality indicator. Descriptive characteristics of the patients and providers can be found in Supplementary Tables 1 and 2, located in Additional File 2. Individuals in both groups were asked to independently endorse items from the PETS that reflect the “most important issues or concerns about self-management and healthcare that *a healthcare provider should know about*.” Item endorsement frequencies were compiled into a report that was provided to a panel of six co-investigators of the project representing expertise in internal medicine, nursing, and health-services research. The panel reviewed the results of the patient and provider winnowing exercises (Supplementary Table 3 in Additional File 2) as well as additional available data on the PETS at an in-person meeting held in May 2017. Rules were established a priori by the panel to guide selection of items for the draft version of the measure (see Additional File 1).

Upon reviewing the data, the panel agreed to include 34 of the original 60 items for the draft Brief PETS quality measure. This shorter version of the original PETS consisted of the following content domains: medical information, medication taking, medication side-effect bother, medical appointments, monitoring health, diet, exercise/physical therapy, relationships with others, medical/healthcare expenses, difficulty with healthcare services, role/social activity limitations and physical/mental exhaustion due to self-management. The items selected for inclusion are identified in Supplementary Table 3 located in Additional File 2.

### Phase II – pilot testing and validation of the brief PETS-quality measure

## Methods

### Sample and study design

We pilot tested the newly-derived Brief PETS-Quality Measure (hereafter, “Brief PETS”) in a prospective study of 400 MCC outpatients. Two hundred patients were recruited from the general-internal medicine clinic of the Hennepin County Medical Center (HCMC: Minneapolis, Minnesota, USA). HCMC is the state of Minnesota’s largest safety-net hospital, providing care for many low-income, uninsured, and vulnerable persons living in urban Minneapolis and surrounding areas. Eligible patients were at least 21 years old, could comprehend English, had ≥2 diagnosed chronic medical conditions requiring self-management (medical record confirmed), and had regularly scheduled appointments with a clinic provider (e.g., about every 6 months). Eligible patients were those who had received an International Classification of Diseases (ICD) diagnostic code from one of their health care providers for one or more of 20 chronic conditions identified by the Department of Health and Human Services as public health priorities of the nation [35, 36]. Furthermore, patients with ICD codes for anxiety, hearing problems, vision problems, irritable bowel/Crohn’s disease, atopic dermatitis/psoriasis, back problems, or headaches were also included as these conditions were identified by our clinical co-investigators (ML, DB, CV, ER, and MF) as having high treatment burden. Patients with an ICD code of a severe cognitive impairment (e.g., dementia) or other conditions (e.g., psychoses) that might make it difficult to understand and complete a survey were ineligible. A total of 335 patients meeting the eligibility criteria were initially screened and approached. There were no differences in gender or age between the 200 enrolled patients and the 135 unenrolled patients. However, enrolled patients had slightly more diagnosed conditions (*M* = 5.4) than unenrolled patients (*M* = 4.7) (*P* < .01). Participation rate of those initially screened was 60% (200/335).

Eligible patients were identified by weekly reports sent to study staff of upcoming patient appointments. Study staff made phone calls to eligible patients to alert them of the opportunity and allow time for the consent process prior to their appointment. The study staff met with patients in the clinic waiting area prior to the appointment to confirm eligibility, explain the study, and orally consent those interested in participating. Those agreeing to participate completed a survey battery that included the Brief PETS and other measures before seeing their provider. Upon completion of this baseline assessment, the staff person informed the patient that they would be contacted again prior to a future appointment to complete a follow-up survey. The follow-up survey was completed in clinic within 6 to 12 months of the baseline survey prior to a scheduled appointment with a provider. A range of time was specified for the follow-ups to allow for individual variability in appointment scheduling and to more closely reflect actual clinical care. Patients received a $5 gift card as compensation for completing each survey. This aspect of the pilot test was approved by the Hennepin Healthcare IRB (HSR #17–4404).

The other 200 patients for this pilot test were drawn from a previous, separate prospective survey study of the full PETS measure conducted in southeast Minnesota (USA). Details of the entire cohort can be found in Eton et al. [30]. In this prospective study, the resources of the Rochester Epidemiology Project (REP) [37] were used to identify a sample of adults living with MCCs in Olmsted County, Minnesota between July 1, 2015 and June 30, 2016. The REP electronically links medical records of local healthcare providers for almost the entire population of Olmsted County, Minnesota [38]. The same set of chronic conditions that determined the HCMC clinic sample (identified through ICD codes) were used to determine eligibility for this community sample. This cohort completed survey batteries including the full PETS and other measures at a baseline assessment and 6, 12, and 24 months post baseline. For the present analysis, a random sample of 200 patients from this cohort with completed surveys at both baseline and 6-month follow-up was drawn after matching to the HCMC patients on gender and number of diagnosed conditions, in order to have equal representation of responses across these variables. The REP sample was drawn *after* data collection at HCMC was complete in order to determine the appropriate follow-up assessment to use from the REP survey study. The mean follow-up timing for the HCMC clinic patients was 7.8 months post baseline; hence, we selected the closest available follow-up assessment from the REP survey (i.e., 6 months). We were able to combine data from the REP and HCMC samples for this analysis because the Brief PETS is a subset of the full PETS and several of the same measures were completed by both sets of patients. This aspect of the pilot test was approved by the Mayo Clinic (14–008629) and Olmsted Medical Center (022-OMC-16) IRBs, institutional co-administrators of the REP.

### Survey

The survey battery given to HCMC patients at both baseline and follow-up consisted of the pilot 34-item Brief PETS, three items from the Centers for Disease Control and Prevention’s (CDC) Healthy Days measure, and a single-item self-report measure of medication adherence. The Healthy Days items ask respondents to report on: (a) the number of poor physical health days in the last 30 days, (b) the number of poor mental health days in the last 30 days, and (c) the number of days of limited activity due to poor physical or mental health in the last 30 days. This measure has been found to be a reliable and valid indicator of health and functional status in people with chronic illnesses [39, 40]. Medication adherence was assessed by the following question: “In a typical week, how close do you come to following your doctor’s recommendations about medications? (always take all of my medications, usually take all of my medications [80% of the time], sometimes take all of my medications [<80% of the time])” [41]. The item has good predictive validity [41] and has been shown to be associated with treatment burden [18]. Demographic characteristics (e.g., age, race/ethnicity, marital and education status, and income) were captured in the baseline survey. In the follow-up survey, two additional questions were included to determine the feasibility and acceptability of the Brief PETS: “How willing are you to complete these questions as a regular part of your visits with your medical providers? (not at all, somewhat, very)” and “Would you like to have your responses shared with your medical providers? (yes, no, do not care one way or the other).”

The Brief PETS, the CDC Healthy Days measure, the medication adherence item, and demographic characteristics were extracted from the survey batteries administered to the REP cohort at baseline and 6-month’s follow-up. Additional measures collected in the REP cohort included scales from the Patient Assessment of Chronic Illness Care (PACIC), a measure of the perceived quality of care received for chronic illness [42]. For this analysis, we used the PACIC’s problem/solving contextual and follow-up coordination scales as these two scales assess activities that form the core of patient-centered self-management support [42]. Finally, a separate investigator-generated item was used to determine whether the respondent had a set routine for all of their self-management (yes/no).

For both the HCMC and REP cohorts, chronic condition diagnoses and gender of the respondent were extracted from the electronic medical record.

### Brief PETS scaling and scoring

We hypothesized scaling of Brief PETS domains based on a recent confirmatory factor analysis (CFA) of the full 60-item PETS (manuscript submitted for publication). This CFA of the full PETS supported scaling of the following multi-item domains: medical information, taking medications, medical appointments, monitoring health, diet, exercise/physical therapy, interpersonal challenges, medical/healthcare expenses, difficulty with healthcare services, role/social activity limitations, and physical/mental exhaustion. Two aggregate index scores were supported by higher-order factor modeling corresponding to aspects of “workload” and “impact.” Workload is an aggregate of the medical information, medications, medical appointments, and monitoring health domains, i.e., domains assessing the “work” associated with treatment and self-management. Impact is an aggregate of the role/social activity limitations and physical/mental exhaustion domains, i.e., domains assessing the “impact” of treatment and self-management on well-being. Overall model fit was good, exceeding published criterion benchmarks [43] with Comparative Fit Index (CFI) = 0.987 (criterion: ≥ 0.95), Root Mean Square Error of Approximation (RMSEA) = 0.03 (criterion: ≤ 0.06), and standardized root mean square residual (SRMR) = 0.06 (criterion: ≤ 0.08). All item factor loadings were ≥ 0.60 supporting the hypothesized domains (manuscript submitted for publication). To verify this domain structure for the Brief PETS, we conducted a CFA on the 400 baseline respondents from the present study (data not shown). Overall model fit was good and exceeded benchmarks with CFI = 0.991, RMSEA = 0.05, and SRMR = 0.06. All factor loadings including those for the workload and impact second-order factors were ≥ 0.60 supporting the hypothesized scaling for the Brief PETS.

Standard PETS scoring was used to derive scores [18, 30]. Missing responses to PETS items can occur when the issue queried is not applicable to the respondent. To handle this, aggregated scale scores are prorated for missing data as long as fewer than 50% of the scale items are missing (equivalent to replacing with the mean of the non-missing items of the scale). The workload and impact index scores are calculated as the mean score of the contributing scales as long as > 50% of those scales are non-missing. All PETS scores use the same 0 to 100 metric with a higher score indicating more treatment burden (see Additional File 3 for the Brief PETS vs. 1.0). To simplify reporting, in this analysis we report the findings for the workload and impact indexes and five other burden domains: diet, exercise/physical therapy, medical expenses, difficulty with healthcare services, and medication side-effects bother (single item). We exclude the interpersonal challenges domain from this report as we have recently re-classified it as a social moderator of treatment burden rather than an indicator of it.

### Analyses

Frequency distributions, means and standard deviations (SD) were used to describe the study sample and characterize Brief PETS scores at baseline. Cronbach’s alpha coefficients were calculated to determine internal consistency reliability of all multi-item domain scales. Acceptable reliability is indicated by an alpha > 0.70 [44]. Spearman rank-order correlation coefficients (rho) were used to determine the association of Brief PETS scores with other factors that have been found to be associated with treatment burden in other studies. Prior studies, including ones using the PETS and other measures of treatment burden, have found higher treatment burden to be associated with being younger [17, 45], having more diagnosed conditions [16, 17, 45], more financial difficulties [16, 18, 45], worse perceived health/well-being [16–18], and lower quality of care [16–18]. Hence, we hypothesized that higher Brief PETS scores (i.e., more treatment burden) will be associated with younger age, more diagnosed conditions, lower income, poorer health/functional status (CDC Healthy Days), and lower quality of care (PACIC) at baseline. Significant correlations of non-trivial magnitude will support validity of the Brief PETS. Cohen’s benchmarks for a small (0.10), moderate (0.30), and large (0.50) correlation magnitude were applied [46]. The power to detect a small-to-moderate correlation of 0.20 based on a sample size of 400 (two-tailed test) at an alpha of 0.05 is 98%.

Known-groups validity was determined by independent-samples t-tests comparing distinct groups at baseline and follow-up. Consistent with findings of prior studies of treatment burden, we hypothesized that higher treatment burden (higher Brief PETS scores) will be associated with not being married (vs. being married) [29, 45, 47], having no more than a high school education (vs. being college-educated) [16, 29], not having a health self-management routine (vs. having one) [16, 29], and being less adherent to taking recommended medications (vs. being more adherent) [18, 19, 30]. For medication adherence, follow-up Brief PETS scores were compared between those with “optimal or improving” status (i.e., consistently good or improving medication adherence from baseline to follow-up) versus “suboptimal or worsening” status (i.e., consistently poor or worsening medication adherence from baseline to follow-up). Alpha for all t-tests was set at .05 (two-tailed). Effect sizes of between-group differences were calculated as Cohen’s *d*, i.e., the group mean difference divided by the pooled within-group SD, with *d* = 0.2 indicating a small effect, *d* = 0.5 indicating a medium effect, and *d* = 0.8 indicating a large effect [46]. Assuming 200 per group at baseline (total *N* = 400), the power to detect a small-to-medium effect size of 0.3 between two groups with independent-samples t-test assuming equal variances is 85% (two-tailed test and alpha of 0.05).

Responsiveness analyses determine whether changes in Brief PETS scores over time (i.e., change scores) coincide with changes in health/functional status. We defined “declining” and “improving” health status groups by comparing respondents’ baseline and follow-up responses to the three CDC Healthy Days measure items (i.e., physical health, mental health, and activity limitations). For each item, declining health was indicated by an *increase* in the number of unhealthy days reported from baseline to follow-up; improving health was indicated by a *decrease* in the number of unhealthy days reported from baseline to follow-up. Brief PETS change scores (Follow-up – baseline) were compared between the declining and improving health status groups using independent-samples t-tests (alpha = .05, two-tailed). Consistent with other studies of treatment burden [16, 17, 30], we hypothesized a worsening of Brief PETS scores (i.e., more burden) in those declining in health/functional status and improving of Brief PETS scores (i.e., less burden) in those improving in health/functional status. Effect size associated with the Brief PETS change score within each health status group was calculated as the standardized response mean (SRM), i.e., the ratio of the mean within-group change to the standard deviation of the change scores [48]. The following benchmarks are used for the SRM: < 0.20 (trivial), 0.20–0.49 (small), 0.50–0.79 (medium), and ≥ 0.80 (large) [46, 49]. Assuming 330 completed follow-up surveys are available for analysis (130 from HCMC [65% response] and 200 from REP) and 165 per group, the power to detect a small effect size of 0.3 between groups with independent-samples t-test assuming equal variances is 78% (two-tailed test and alpha of 0.05).

Endorsement frequencies were checked for the items assessing feasibility and acceptability of the Brief PETS that were queried at follow-up in the HCMC patient cohort. All analyses were conducted in IBM SPSS Statistics for Windows (Version 25.0, Armonk, NY: IBM Corp.).

## Results

### Sample characteristics

Descriptive and clinical characteristics of the sample appear in Table 1. The full sample combines the 200 patients recruited from the HCMC primary care clinic with the 200 patients drawn from the REP cohort matched on gender and number of diagnosed conditions. Mean age of the full sample of 400 patients was 57.9 years (SD = 12.8), 50% were female, and 38% were married or living with a partner. Race was 48% White/Caucasian, 37% Black/African American, and the rest of mixed or other races. Fifty-seven percent were college-educated and 51% reported an annual income of less than $20,000. Median number of diagnosed chronic conditions was 5.0 (range: 2–13) and the most frequent diagnoses were hypertension, diabetes, low back disorder, depression, hyperlipidemia, arthritis, and substance abuse. There tended to be more mental health conditions such as depression, anxiety, and substance abuse represented in the HCMC cohort. While the full sample was used in the validation analyses to enhance generalizability, compared to the REP cohort the HCMC patients were less adherent to taking recommended medications and reported poorer physical and mental health in the past 30 days (*P*s < .001). Follow-up surveys were available on 83% of the baseline sample (132 HCMC and 200 REP).

**Table 1:** Descriptive and clinical characteristics at baseline

	Full sample (N = 400)	HCMC sample (N = 200)	REP sample (N = 200)
**Age:** Mean (SD)	57.9 (12.8)	54.2 (9.6)	61.6 (14.5)
**Female:** N (%)	199 (50%)	99 (50%)	100 (50%)
**Race:** N (%)			
White / Caucasian	191 (48%)	33 (17%)	158 (79%)
Black / AA	147 (37%)	131 (66%)	16 (8%)
Mixed race	18 (5%)	12 (6%)	6 (3%)
Native American	16 (4%)	15 (8%)	1 (< 1%)
Asian	11 (3%)	2 (1%)	9 (5%)
Other	12 (3%)	7 (4%)	5 (3%)
Unknown	5 (1%)	0 (0%)	5 (3%)
**Hispanic ethnicity:** N (%)	14 (4%)	7 (4%)	7 (4%)
**Married / living with partner:** N (%)	152 (38%)	26 (13%)	126 (63%)
**College educated:** N (%)	229 (57%)	77 (39%)	152 (76%)
**Annual income:** N (%)			
< $20,000	203 (51%)	170 (85%)	33 (17%)
$20,000 - $39,000	60 (15%)	24 (12%)	36 (19%)
$40,000 - $59,000	35 (9%)	2 (1%)	33 (17%)
$60,000 - $79,999	30 (8%)	0 (0%)	30 (15%)
$80,000 - $99,000	19 (5%)	0 (0%)	19 (10%)
$100,000 or more	39 (10%)	3 (2%)	36 (18%)
Missing	14 (4%)	1 (< 1%)	13 (7%)
**Number of diagnoses:** Median (range)	5.0 (2–13)	5.0 (2–13)	5.0 (2–11)
**Types of conditions:** N (%)			
Hypertension	243 (61%)	127 (64%)	116 (58%)
Diabetes (Type 1 or 2)	215 (54%)	113 (57%)	102 (51%)
Low back disorder^a^	208 (52%)	106 (53%)	102 (51%)
Depression	208 (52%)	124 (62%)	84 (42%)
Hyperlipidemia	190 (48%)	75 (38%)	115 (58%)
Arthritis	167 (42%)	68 (34%)	99 (50%)
Substance abuse	144 (36%)	122 (61%)	22 (11%)
Anxiety	137 (34%)	86 (43%)	51 (26%)
Cancer	74 (19%)	22 (11%)	52 (26%)
Cardiac arrhythmia	74 (19%)	28 (14%)	46 (23%)
Vision problems	70 (18%)	0 (0%)	70 (35%)
Coronary artery disease	64 (16%)	22 (11%)	42 (21%)
Asthma	58 (15%)	58 (29%)	0 (0%)
Headache	55 (14%)	38 (19%)	17 (9%)
COPD	49 (12%)	25 (13%)	24 (12%)
Chronic kidney disease	47 (12%)	23 (12%)	24 (12%)
Hearing problems	34 (9%)	0 (0%)	34 (17%)
Hepatitis	26 (7%)	20 (10%)	6 (3%)
Congestive heart failure	24 (6%)	12 (6%)	12 (6%)
Osteoporosis	18 (5%)	4 (2%)	14 (7%)
Crohn’s disease	11 (3%)	0 (0%)	11 (6%)
Psoriasis	10 (3%)	0 (0%)	10 (5%)
HIV	2 (< 1%)	2 (1%)	0 (0%)
**Weekly medication adherence:** N (%)			
Always take all my medications	275 (69%)	120 (60%)	155 (78%)
Usually take all my medications	95 (24%)	57 (29%)	38 (19%)
Sometimes take all my medications	22 (6%)	18 (9%)	4 (2%)
Missing	8 (2%)	5 (3%)	3 (2%)
**Number of poor physical health days in past 30 days:** Mean (SD)^b^	10.7 (10.2)	13.3 (10.0)	8.1 (9.7)
**Number of poor mental health days in past 30 days:** Mean (SD)^b^	9.6 (10.1)	11.9 (10.2)	7.3 (9.5)
**Number of days of limited activity due to poor physical or mental health in past 30 days:** Mean (SD)^b^	8.5 (10.0)	11.6 (10.3)	5.4 (8.6)

^a^Includes osteopathic conditions such as disc displacement/degeneration, spondylosis, spinal stenosis, sciatica, and post-laminectomy syndromes. ^b^Centers for Disease Control and Prevention, Healthy Days measure. *SD* standard deviation, *HCMC* Hennepin County Medical Center (Minneapolis, Minnesota, USA), *REP* Rochester Epidemiology Project (Olmsted County, Minnesota, USA)

### Reliability of brief PETS scales and baseline scale means

As shown in Table 2, among the ten multi-item Brief PETS scales, nine exceeded the threshold for acceptable internal consistency reliability (Cronbach’s alpha > 0.70) [44]. Reliability for the 2-item exercise/physical therapy scale was below this threshold at alpha = 0.62. Table 2 also shows the mean Brief PETS scores at baseline for the full sample of 400 and the HCMC and REP cohorts separately. Overall, mean Brief PETS scores indicated higher treatment burden in the HCMC cohort than the REP cohort (*P*s < .01), with the exception of the medication side-effects bother scale (unadjusted analyses). It is important to note that these two cohorts are comparable on gender and number of chronic conditions, the two variables upon which they were matched.

**Table 2:** Brief PETS scores (mean and SD) at baseline and Cronbach’s alphas for multi-item scales

Brief PETS score^a^	Full sample (N = 400)	HCMC sample (N = 200)	REP sample (N = 200)	*P*-value^e^
Workload summary index^b^	30.4 (19.8)	36.8 (20.3)	24.2 (17.3)	< .001
Medical information (alpha = 0.89)	32.5 (21.0)	37.2 (22.0)	27.8 (18.8)	< .001
Medications (alpha = 0.87)	22.1 (23.9)	29.7 (26.7)	14.8 (18.0)	< .001
Medical appointments (alpha = 0.74)	27.6 (24.6)	33.7 (26.4)	21.6 (21.2)	< .001
Monitoring health (alpha = 0.89)	39.8 (27.7)	45.9 (27.4)	33.5 (26.5)	< .001
Impact summary index^c^	34.0 (25.9)	42.8 (25.3)	25.5 (23.5)	< .001
Role activity limitations (alpha = 0.93)	30.1 (30.2)	39.3 (31.1)	21.2 (26.4)	< .001
Physical / mental exhaustion (alpha = 0.92)	38.0 (26.7)	46.5 (26.0)	29.8 (24.8)	< .001
Diet^d^ (alpha = 0.72)	52.0 (24.7)	55.8 (23.4)	45.6 (25.5)	< .01
Exercise / physical therapy^d^ (alpha = 0.62)	59.9 (25.8)	66.2 (24.0)	51.5 (25.8)	< .001
Medical expenses (alpha = 0.87)	46.1 (27.0)	50.0 (26.2)	42.4 (27.3)	< .01
Difficulty with healthcare services (alpha = 0.72)	43.3 (26.2)	51.8 (25.9)	33.5 (23.0)	< .001
Medication side-effects bother (alpha: NA)	21.8 (27.4)	24.4 (28.1)	19.2 (26.6)	NS

^a^Higher PETS score = more burden (0 = lowest burden; 100 = highest burden). ^b^Aggregated mean of scores in the medical information, medications, medical appointments, and monitoring health scales. Calculated when > 50% of the four constituent scales are non-missing. ^c^Aggregated mean of scores in the role/social activity limitations and physical/mental exhaustion scales. Calculated when > 50% of the two constituent scales are non-missing (i.e., when both scales are non-missing). ^d^Yes/no screener used for the diet and exercise / physical therapy domains. ^e^*P*-value associated with t-test comparison of Brief PETS scores between HCMC and REP samples. *SD* standard deviation, *NA* not applicable, *NS* not significant, *HCMC* Hennepin County Medical Center (Minneapolis, Minnesota, USA), *REP* Rochester Epidemiology Project (Olmsted County, Minnesota, USA)

### Correlations of brief PETS with demographic, clinical, and quality of care indicators

As shown in Table 3 and as hypothesized, younger age and lower income were each associated with higher treatment burden across all Brief PETS scores, with correlation sizes (rho) ranging from small (− 0.16) to moderate (− 0.41). Total number of diagnosed chronic conditions was unrelated to Brief PETS treatment burden scores. CDC Healthy Days measure reports of poorer physical health, poorer mental health, and greater limitations in activities in the past 30 days were each associated significantly with higher PETS treatment burden scores, with correlation sizes ranging from small (0.19) to large (0.66). Finally, certain aspects of treatment burden were significantly associated with the Problem solving/Contextual scale of the PACIC (REP cohort only). Higher burden of workload, medical expenses, difficulty with healthcare services, and medication side-effect bother were associated with less provider consideration of the patient’s social and cultural environment when making a treatment plan, with these correlations ranging in size from small (− 0.17) to moderate (− 0.32). None of the Brief PETS scores were significantly correlated with the Follow-up Coordination scale of the PACIC.

**Table 3:** Correlations of Brief PETS scores with demographic, clinical, and quality of care indicators at baseline

Brief PETS score^a^	Age	Total no. of conditions	Annual income^d^	No. of poor physical health days in last 30^e^	No. of poor mental health days in last 30^e^	No. of days limited by poor physical/mental health in last 30^e^	PACIC Problem solving / contextual^f^	PACIC Follow-up coordination^f^
**Workload index**^b^	−0.28	−0.03	−0.34	0.43	0.48	0.53	− 0.22	− 0.12
	*P* < .001	NS	*P* < .001	*P* < .001	*P* < .001	*P* < .001	*P* < .005	NS
**Impact index**^c^	− 0.37	0.07	− 0.41	0.61	0.61	0.66	− 0.12	0.00
	*P* < .001	NS	*P* < .001	*P* < .001	*P* < .001	*P* < .001	NS	NS
**Diet**	−0.17	−0.04	− 0.16	0.19	0.23	0.19	−0.18	−0.10
	*P* < .01	NS	*P* < .05	*P* < .005	*P* < .001	*P* < .005	NS	NS
**Exercise / physical therapy**	−0.26	0.07	−0.35	0.47	0.42	0.42	0.06	0.15
	*P* < .001	NS	*P* < .001	*P* < .001	*P* < .001	*P* < .001	NS	NS
**Medical expenses**	−0.26	0.02	−0.26	0.37	0.37	0.37	−0.17	−0.10
	*P* < .001	NS	*P* < .001	*P* < .001	*P* < .001	*P* < .001	*P* < .05	NS
**Difficulty with healthcare services**	− 0.23	−0.02	− 0.34	0.27	0.25	0.27	−0.32	−0.13
	*P* < .001	NS	*P* < .001	*P* < .001	*P* < .001	*P* < .001	*P* < .001	NS
**Medication side-effect bother**	− 0.17	0.04	− 0.19	0.31	0.25	0.29	−0.17	−0.11
	*P* < .005	NS	*P* < .001	*P* < .001	*P* < .001	*P* < .001	*P* < .05	NS

^a^Higher PETS score = more burden. ^b^Aggregated mean of medical information, medications, medical appointments, and monitoring health scales. ^c^Aggregated mean of role/social activity limitations and physical/mental exhaustion scales. ^d^From < $20,000/year (code = 1) to ≥ $100,000/year (code = 6). ^e^Centers for Disease Control and Prevention, Healthy Days measure. ^f^Patient Assessment of Chronic Illness Care: Higher score indicates better quality of care in the indicated domain. Scale administered in the REP sample only (*N* = 200)

### Known-groups comparisons of brief PETS scores at baseline

Comparisons of Brief PETS scores across groups defined by marital and education status appear in Table 4. As hypothesized, unmarried patients reported significantly higher treatment burden than married/partnered patients, in 6 of 7 Brief PETS scores. Effect sizes (*d*) of the mean differences ranged from small (0.25) to moderate (0.54). Also as hypothesized, patients with less formal education (no more than high school) reported significantly higher treatment burden than patients with a college education, in 6 of 7 Brief PETS scores. Effect sizes of these mean differences also ranged from small (0.31) to moderate (0.52). There were no differences in the Brief PETS medication side-effect bother score by either marital or education status. Also in Table 4 are results of comparisons of Brief PETS scores by patient endorsement of having versus not having a set self-management routine (REP cohort only). When compared to patients who have a self-management routine, those who do not reported higher workload, impact, and diet burden with effect sizes of these group differences being moderate (0.54 to 0.58).

**Table 4:** Known-group comparisons of Brief PETS scores at baseline

Brief PETS score^a^	Marital status		Education status		Self-management routine?^e^	
	Unmarried *N* = 152	Married / partnered *N* = 240	HS graduate or less *N* = 165	College-educ. *N* = 229	No *N* = 48	Yes *N* = 152
**Workload index**^b^						
*Mean (SD)*	34.7 (21.0)	24.7 (16.1)	35.6 (19.7)	27.3 (19.6)	31.3 (19.3)	21.9 (16.1)
*T (Effect size)*	*T*(386) = 5.01, *P* < .001 (*d* = 0.51)		*T*(388) = 4.19, *P* < .001 (*d* = 0.42)		*T*(197) = 3.37, *P* < .005 (*d* = 0.54)	
**Impact index**^c^						
*Mean (SD)*	39.5 (25.3)	26.4 (24.9)	41.8 (24.8)	28.9 (25.4)	35.9 (24.8)	22.2 (22.2)
*T (Effect size)*	*T*(384) = 5.00, *P* < .001 (*d* = 0.51)		*T*(386) = 4.97, *P* < .001 (*d* = 0.50)		*T*(198) = 3.64, *P* < .001 (*d* = 0.58)	
**Diet**^d^						
*Mean (SD)*	55.7 (24.6)	44.1 (22.6)	57.3 (24.2)	48.1 (23.7)	57.4 (25.7)	42.8 (24.8)
*T (Effect size)*	*T*(249) = 3.60, *P* < .001 (*d* = 0.47)		*T*(249) = 3.03, *P* < .005 (*d* = 0.38)		*T*(92) = 2.23, *P* < .05 (*d* = 0.57)	
**Exercise / Phys. Ther.**^d^						
*Mean (SD)*	65.0 (24.9)	50.9 (25.3)	64.6 (24.7)	56.2 (26.2)	56.0 (27.6)	50.3 (25.4)
*T (Effect size)*	*T*(276) = 4.51, *P* < .001 (*d* = 0.54)		*T*(278) = 2.73, *P* < .01 (*d* = 0.33)		*T*(119) = 0.98, NS (*d* = 0.22)	
**Medical expenses**						
*Mean (SD)*	48.8 (26.7)	42.0 (26.3)	51.0 (26.1)	42.7 (26.9)	46.6 (28.9)	41.2 (26.8)
*T (Effect size)*	*T*(356) = 2.38, *P* < .05 (*d* = 0.25)		*T*(358) = 2.91, *P* < .005 (*d* = 0.31)		*T*(186) = 1.14, NS (*d* = 0.20)	
**Diff. healthcare services**						
*Mean (SD)*	47.5 (27.0)	37.5 (22.8)	50.9 (26.5)	37.4 (24.2)	35.2 (20.9)	32.8 (23.7)
*T (Effect size)*	*T*(286) = 3.24, *P* < .001 (*d* = 0.39)		*T*(289) = 4.53, *P* < .001 (*d* = 0.52)		*T*(134) = 0.53, NS (*d* = 0.10)	
**Med. side-effect bother**						
*Mean (SD)*	23.3 (28.4)	20.4 (26.1)	22.5 (27.8)	21.7 (27.4)	22.9 (26.2)	18.0 (26.6)
*T (Effect size)*	*T*(384) = 1.01, NS (*d* = 0.11)		*T*(386) = 0.28, NS (*d* = 0.03)		*T*(197) = 1.11, NS (*d* = 0.32)	

^a^Higher PETS score = more burden. ^b^Mean of medical information, medications, medical appointments, and monitoring health scales. ^c^Mean of role/social activity limitations and physical/mental exhaustion scales. ^d^Yes/no screener used for the diet and exercise / physical therapy domains. ^e^Question administered in the REP sample only (*N* = 200). Sample sizes may fluctuate per analysis due to missing data on PETS scales

### Follow-up brief PETS scores by prospective medication adherence status

**Fig. 2:**
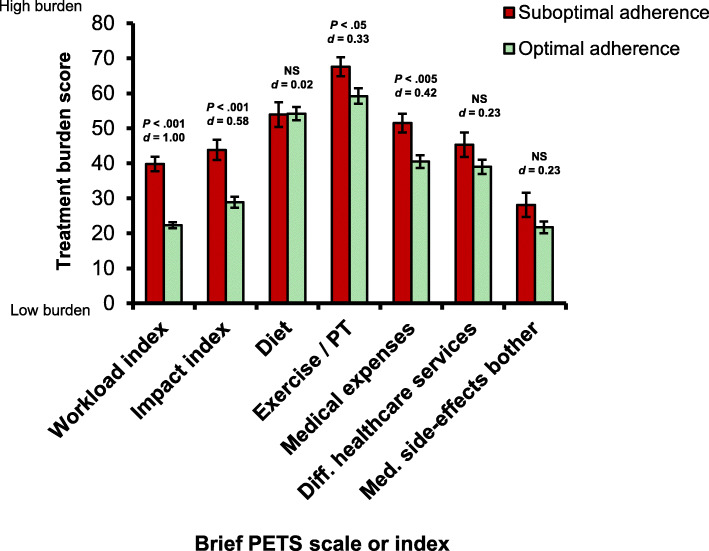
Mean follow-up treatment burden score by self-reported medication adherence. Legend: PETS: Patient Experience with Treatment and Self-management; Exercise / PT: Exercise / Physical Therapy; NS: Not significant

At follow-up, patients who reported suboptimal medication adherence over time reported significantly greater burden in workload, impact, exercise/physical therapy, and medical expenses compared to those who reported having optimal medication adherence over time (see Fig. 2). Effect sizes of these group differences ranged from small (0.33) to large (1.00). There were no significant differences in diet burden, difficulty with healthcare services, or medication side-effect bother across medication adherence.

### Responsiveness of brief PETS to changes in health status

Table 5 shows the results of analyses of Brief PETS change scores across health status groups defined by the three CDC Healthy Days items. Patients reporting declines in *physical health* had significant worsening of burden in workload, impact, and exercise/physical therapy when compared to those reporting improvements in physical health. Patients reporting declines in *mental health* had significant worsening of burden in workload, impact, diet, and difficulty with healthcare services when compared to those reporting improvements in mental health. Finally, patients reporting greater *activity limitations* due to poor physical or mental health had significant worsening of burden in workload, impact, exercise/physical therapy, and difficulty with healthcare services when compared to those reporting fewer activity limitations. Across all comparisons declining health status was always associated with an increase in reported treatment burden, whereas improving health status was always associated with a decrease in reported treatment burden. The within-group SRMs (absolute value) were generally small, ranging from 0.04 to 0.44, with 73% > 0.20 or non-trivial. No significant health-status group differences in treatment burden change were found for medical expenses burden or medication side-effect bother.

**Table 5:** Responsiveness of Brief PETS to changes in health status from baseline to follow-up assessment

Brief PETS change score^a^	Change in physical health status in past 30 days^e^		Change in mental health status in past 30 days^e^		Change in activity limitation status in past 30 days^e^	
	Declining health *N* = 113 (Chg. unhealthy days mean: + 7.7)	Improving health *N* = 115 (Chg. unhealthy days mean: − 8.3)	Declining health *N* = 106 (Chg. unhealthy days mean: + 7.8)	Improving health *N* = 90 (Chg. unhealthy days mean: − 8.5)	Declining health *N* = 106 (Chg. unhealthy days mean: + 8.9)	Improving health *N* = 99 (Chg. unhealthy days mean: − 8.3)
**Δ Workload index**^b^						
*Mean (SD)*	0.9 (14.0)	−3.9 (15.0)	0.5 (12.7)	−4.1 (15.7)	1.4 (13.8)	−6.5 (14.9)
*SRM* (abs. value)	0.06	0.26	0.04	0.26	0.10	0.44
*T* (between group)	*T*(224) = 2.44, *P* < .05		*T*(192) = 2.24, *P* < .05		*T*(201) = 3.94, *P* < .001	
**Δ Impact index**^c^						
*Mean (SD)*	6.7 (24.2)	−5.1 (21.0)	8.0 (24.0)	−6.1 (20.9)	7.2 (24.1)	−7.4 (21.1)
*SRM* (abs. value)	0.28	0.24	0.33	0.29	0.30	0.35
*T* (between group)	*T*(224) = 3.92, *P* < .001		*T*(192) = 4.34, *P* < .001		*T*(200) = 4.57, *P* < .001	
**Δ Diet**^d^						
*Mean (SD)*	0.0 (27.7)	−2.7 (24.6)	5.4 (21.4)	−6.2 (25.5)	1.7 (22.4)	−5.0 (25.6)
*SRM* (abs. value)	0.00	0.11	0.25	0.24	0.08	0.20
*T* (between group)	*T*(102) = 0.52, NS		*T*(90) = 2.39, *P* < .05		*T*(91) = 1.35, NS	
**Δ Exercise / Phys. Ther.**^d^						
*Mean (SD)*	6.4 (30.8)	−3.9 (23.6)	4.6 (24.9)	−4.1 (29.3)	8.9 (26.5)	−8.8 (25.0)
*SRM* (abs. value)	0.21	0.17	0.18	0.14	0.34	0.35
*T* (between group)	*T*(115) = 2.04, *P* < .05		*T*(113) = 1.72, NS		*T*(107) = 3.56, *P* < .001	
**Δ Medical expenses**						
*Mean (SD)*	0.0 (25.8)	−5.5 (24.6)	−1.34 (25.9)	−7.5 (24.4)	− 1.4 (28.5)	−8.3 (23.4)
*SRM* (abs. value)	0.00	0.22	0.05	0.31	0.05	0.35
*T* (between group)	*T*(191) = 1.51, NS		*T*(166) = 1.56, NS		*T*(176) = 1.75, NS	
**Δ Diff. healthcare services**						
*Mean (SD)*	4.4 (28.9)	−2.2 (25.7)	3.2 (25.4)	−6.3 (25.4)	5.0 (27.5)	−5.6 (26.3)
*SRM* (abs. value)	0.15	0.09	0.13	0.25	0.18	0.21
*T* (between group)	*T*(132) = 1.38, NS		*T*(124) = 2.07, *P* < .05		*T*(127) = 2.24, *P* < .05	
**Δ Med. side-effect bother**						
*Mean (SD)*	5.5 (25.5)	2.6 (33.0)	4.5 (28.5)	7.1 (29.8)	0.2 (31.0)	4.5 (31.0)
*SRM* (abs. value)	0.22	0.08	0.16	0.24	0.01	0.15
*T* (between group)	*T*(221) = 0.73, NS		*T*(191) = 0.61, NS		*T*(200) = 0.99, NS	

^a^Follow-up – baseline PETS score: positive PETS change score indicates an increase in burden over time; negative PETS change score indicates a decrease in burden over time. ^b^Mean of medical information, medications, medical appointments, and monitoring health scales. ^c^Mean of role/social activity limitations and physical/mental exhaustion scales. ^d^Yes/no screener used for the diet and exercise / physical therapy domains. ^e^From the Centers for Disease Control and Prevention Healthy Days measure. “Declining health” indicates an increase in the number of unhealthy days reported from the baseline to the follow-up assessment. “Improving health” indicates a decrease in the number of unhealthy days reported from the baseline to the follow-up assessment. Sample sizes may fluctuate per analysis due to missing data on PETS scales. SRM: standardized response mean (absolute value)

### Feasibility and acceptability of brief PETS

At the follow-up assessment, among the HCMC clinic patients, the vast majority (91%) endorsed being either very or somewhat willing to complete the Brief PETS at their regular provider visits. Many (50%) preferred to have their responses shared with their providers, while 36% did not care one way or the other. Only a minority (14%) preferred not to share their responses with their providers.

## Discussion

In primary care, there is a critical need to develop and refine clinical pathways that reach beyond a focus on single diseases to ones that appreciate the complexities experienced by patients dealing with multiple chronic conditions (MCCs) [4]. Integral to this from a person-centered perspective is achieving greater understanding of the complex nature of living with and caring for MCCs on a day-to-day basis [50]. The most valuable source for such information is the patient. Our PETS measure of treatment burden was derived entirely from input from patients with MCCs, and is intended for use with them [14, 18]. It comprehensively assesses the patient’s experience with all treatments and self-management assigned to them by their healthcare providers, including how difficult it is to maintain such self-care and the impact that it has on overall well-being. The aim of this study was to create a shorter version of the PETS, one tailored to the measurement of quality in primary care. This would represent a unique indicator of quality when compared to other available measures that assess healthcare services. Current models of assessing provider services address the consumers’ experiences with services delivered at the *point of clinical care* which is usually a healthcare facility [11, 42]. No quality measure currently exists that thoroughly addresses how easy or difficult it is to integrate provider prescribed self-management into daily life *outside* of the formal healthcare setting.

We relied on input from patients and healthcare providers to specify a Brief PETS measure for quality assessment. These two groups identified issues from the full (60-item) PETS measure that represent the most important issues of treatment burden that a healthcare provider treating a person with MCCs should know about. These could reflect challenges that make it harder for patients to self-manage their condition, adhere to prescribed regimens, or otherwise impact their well-being and quality of life. Results of this initial vetting were reviewed by an expert panel of nurses, primary care physicians, and health-services research experts, whom also had access to archived PETS data to make final decisions about the content for the pilot Brief PETS measure. The resulting measure was subjected to validation testing in a socio-demographically diverse mix of MCC patients from two different healthcare systems.

Validation testing largely supported the utility of the Brief PETS measure. Most of the multi-item scales (9 of 10) had acceptable internal consistency reliability with alphas ranging from 0.72 to 0.93. Only the exercise/physical therapy scale (alpha = 0.62) fell below acceptable reliability. Overall, internal consistencies of the shortened Brief PETS scales were lower than that of the longer scales of the full PETS measure, all found to be > 0.80 [18, 30]. This is not unexpected as internal consistency is typically lower in shorter scales in comparison to longer scales of the same domain construct as items representative of the underlying construct are removed. Hence, some measurement precision as indicated by internal-consistency reliability is sacrificed in the abbreviated Brief PETS scales in comparison to the longer scales of the full PETS. Supporting validity, Brief PETS scores were associated with age, income, and quality of care in expected ways. Consistent with other studies [16, 45, 51], younger age was associated with more treatment burden, with most of the associations (86%) of a small-to-moderate magnitude (rho = 0.10 to 0.30). This may reflect differences in role responsibilities between younger and older persons or age-related differences in the way in which quality of life is appraised [45]. Lower annual income was associated with more treatment burden, with most of the associations (57%) of a moderate-to-large magnitude (rho = 0.30 to 0.50). This comports with findings from the full PETS where financial difficulties were strongly associated with higher treatment burden scores [18]. Finally, several Brief PETS scores (4 of 7) were associated with the problem solving/contextual scale of the PACIC at a small-to-moderate magnitude level (rho = 0.10 to 0.30), but none were associated with the follow-up care/coordination scale. Small associations of treatment burden and PACIC scores have been observed in other studies [16] and may indicate that while measurement of treatment burden is related to existing assessments of chronic care quality, it is not redundant with them.

Contrary to hypothesis, Brief PETS scores were not associated with the number of diagnosed conditions. The relationship between scores of treatment burden measures and number of diagnoses is mixed with some studies showing a moderate positive relationship [16, 17, 45] and others showing low or no relationship [18, 30, 52]. This may be due to study differences in how diagnosed conditions are captured (self-report vs. record extraction), or clinical and social factors such as the types and severity of diagnosed conditions, the length of time living with the conditions, or the presence of available resources that may lessen burden [30, 47, 52].

Known-groups validity of the Brief PETS was supported in analyses of baseline and follow-up scores. Findings agree with those of other studies using the full PETS and other treatment burden measures. Higher Brief PETS scores at baseline were observed in unmarried compared to married or partnered patients. Qualitative reports suggest that close family members, especially spouses, play a critical role in mitigating treatment burden in those with multimorbidity [47], and a recent survey study of multi-morbid cancer survivors has shown that supportive relationships are associated with lower PETS burden scores [29]. Additionally, higher Brief PETS scores at baseline were observed in those with less formal education (vs. more formal education) and in those who reported that they do not have a routine for all of their self-management (vs. those who do have a routine). These findings replicate those observed in the aforementioned cancer survivor study that used scales of the full PETS measure [29]. They are also consistent with studies showing that higher education attained and knowledge about one’s health and being more proactive about self-care are associated with lower perceptions of treatment burden [16, 19]. Finally, patients who reported suboptimal adherence to recommended medications over time had higher follow-up Brief PETS burden scores compared to those who reported optimal medication adherence over time. This is consistent with findings of studies using the full PETS measure [18, 30] and one other measure of treatment burden [19, 22].

Supportive of responsiveness to change of the Brief PETS, we found several differences in burden change scores from baseline to follow-up when comparing patient groups defined by the CDC’s Healthy Days measure as either declining or improving in health. Generally speaking, patients who reported declines in physical and mental health status and those experiencing more activity limitations had worsening burden scores, whereas those reporting improvements in these health status areas had improving burden scores. This was most consistently observed in the workload and impact burden scores, though also seen in the exercise/physical therapy and difficulties with healthcare services scores. Within-group effect sizes of these burden changes were small, but not trivial. Differences observed were consistent with responsiveness findings of the full PETS measure [30] and two other established measures of treatment burden [16, 17].

Finally, our data support that the Brief PETS is both feasible and acceptable to patients. Among the clinic sub-sample, 91% endorsed a willingness to complete the Brief PETS as part of their regular visits with providers and 86% did not object to having their responses shared with their providers.

### Practical implications: moving toward more “person-centered” quality assessment for people with multi-morbidity

There is a deficiency in quality measurement specific to patients with MCCs [4, 6]. We have attempted to help fill the gap by adapting and validating a brief measure of treatment burden informed by MCC patients and their healthcare providers. It could be argued that a patient’s subjective assessment of treatment burden should not be a standard to hold a healthcare provider to. However, emerging frameworks of “person-centered” healthcare challenge this argument [4, 53]. Many outcomes important to people with complex chronic conditions are too infrequently addressed or even mentioned by health professionals, including maintaining a patient’s independence and reducing the tendency of chronic care management to define people’s lives [53]. As asserted by Valderas and colleagues [4]:

People-centeredness, a core value of health systems, acknowledges that individual service users should be the key stakeholders. Their values, goals, and priorities should shape care delivery and individual care plans, and this should be reflected accordingly in quality indicators … Assessment frameworks that capture patient preferences and values and incorporate patients’ voices in the form of patient-reported experiences and outcomes of care will be critical for making progress towards the achievement of high-performing health systems. (P. 296)

The felt need to control clinical outcome metrics has expanded the requests that providers and health systems make of patients in the interest of improving long-term morbidity and mortality. However, while patients are expected to adapt to the expanding burdens of treatment and care to meet these metrics, it is less clear that providers understand the depth or totality of this burden and its impact on patients [54]. These issues are magnified in people with MCCs who may be asked by different providers to strictly adhere to management guidelines of several diseases simultaneously [55]. Adherence to guideline-recommended care in people with MCCs may be associated with an impractically high level of care complexity, cost, and burden [56]. It is in this context that the Brief PETS could be a useful tool for providing feedback to providers and healthcare systems on patient treatment burden, leading to better alignment between treatment goals and individual preferences.

It would however be inappropriate to measure treatment burden in isolation from other quality indicators of primary care. Thoughtful integration of clinical benchmarks with person-centered indicators like treatment burden is needed to assure providers that optimal clinical outcomes are achieved without sacrificing high-quality, person-centered care. To achieve this, two critical questions must be addressed. Can clinical outcome metrics be modified and made less rigid for those with MCCs to minimize treatment burden and its negative effects? Can the pursuit of metric targets be balanced against the healthcare work required of a patient to achieve them? We believe that ‘yes’ answers to both of these questions will be a positive step toward achieving care that is efficacious and tailored to the needs and values of each patient. There have been recent calls by physicians to integrate considerations of treatment burden into future clinical treatment guidelines [54] as well as to include it as a measure of quality in primary care clinical practice [57, 58]. Quality measures that are more closely aligned with the realities of primary care practice should promote accountable performance and “boost clinicians’ motivation by rewarding them for managing complexity, solving problems, and thinking creatively when addressing the unique circumstances of each patient” (P. 175) [58].

### Study limitations

Our study has limitations. First, as described in Additional File 1 the patients and providers queried in the Phase I winnowing process all came from one healthcare system (HCMC), with the patient sample being one of convenience. This represents a limitation in the sampling that might have impacted item selection, though use of an independent panel to review the results served as a check on representativeness of the item set. Second, generalizability of the findings of the validation test may be limited because responses to some measures were only available in one of the cohorts. Responses to the PACIC measure and the question about the presence/absence of a self-management routine were only available in the REP community-sample cohort. Lower sample sizes may also have resulted in reduced power for these analyses. Third, the exclusion of persons with severe cognitive impairments in both Phases I and II means that we cannot draw any conclusions about the treatment burden of these individuals. Fourth, to obtain Brief PETS scores from the REP cohort, we extracted the appropriate items from the full PETS measure that was administered. It is possible that responses to the Brief PETS items in this cohort may have been influenced by responses to other items in the long-form version (i.e., context effects). Fifth, at this point clinical significance of PETS scores has not been determined, so we cannot conclude with certainty that the between-group differences and changes in burden scores correspond to differences that are clinically meaningful. However, many of the differences and changes observed were within the range of a small to moderate effect size (0.2 to 0.5) which has been shown to correspond to clinically important differences on other well-established PRO measures [59]. Sixth, given that all patients studied had multiple diagnoses, we were not able to compare treatment burden scores across individual medical conditions. Finally, it is noteworthy that there were no differences in medication side-effect bother between any of the discrete groups compared in the pilot test. While further testing is warranted, this single-item scale may lack the sensitivity of the other multi-item scales of the Brief PETS.

## Conclusion

The conceptual framework and content of the PETS treatment burden measure [18, 30] has provided the foundation for deriving a short-form version adapted for the measurement of quality in primary-care settings. Input from primary-care patients and providers helped to isolate treatment burden issues of greatest relevance to the quality of care of patients with complex health situations such as those with MCCs. The Brief PETS measure derived in this study appears to be reliable, valid, and responsive to change over time. At 32 items, the final version of the Brief PETS (see Appendix in Additional File 3) is considerably shorter than the original full PETS measure. We believe that this shorter length will make it an appealing option for busy clinical practices interested in tracking treatment burden in the patients that they see [3], especially practices that care for a large proportion of multi-morbid patients such as those in primary-care internal medicine and family medicine. We are confident that it has relevance beyond the USA given that the PETS conceptual framework has informed the development of treatment burden measures in Europe [17, 31] and cultural/linguistic translations of the full PETS measure are now available in select countries [32] with others forthcoming.

## Supplementary Information

**Additional file 1.** Deriving a brief measure of treatment burden to assess person-centered healthcare quality in primary care.

**Additional file 2.** Supplementary data tables for derivation phase.

**Additional file 3: Appendix:** The Brief Patient Experience with Treatment and Self-management (Brief PETS).

## Data Availability

The datasets generated and/or analyzed during the current study are not publicly available as they are governed by a resource sharing plan of the funded project. Datasets can be made available to interested investigators upon reasonable request and approval of the corresponding author, provided that all conditions of data sharing as stipulated in the resource sharing plan are met. All requests are subject to review by the project principal and co-investigators. The Brief PETS measure along with its scoring are available from the corresponding author.

## Abbreviations

CAHPS: Consumer Assessment of Healthcare Providers and Systems
CDC: Centers for Disease Control and Prevention
CFA: Confirmatory Factor Analysis
CFI: Comparative Fit Index
ES: Effect Size
HCMC: Hennepin County Medical Center (Minneapolis, Minnesota, USA)
ICHOM: International Consortium for Health Outcomes Measurement
IRB: Institutional Review Board
MCC: Multiple Chronic Conditions
PACIC: Patient Assessment of Chronic Illness Care
PETS: Patient Experience with Treatment and Self-management
PREM: Patient-Related Experience Measure
PROM: Patient-Reported Outcome Measure
REP: Rochester Epidemiology Project
RMSEA: Root Mean Square Error of Approximation
SD: Standard Deviation
SRM: Standardized Response Mean
SRMR: Standardized Root Mean Square Residual
