# Supplementary material for: Deriving and validating a brief measure of treatment burden to assess person-centered healthcare quality in primary care: a multi-method study
Source: BMC Fam Pract. 2020 Oct 28;21:221. doi: 10.1186/s12875-020-01291-x (PMC7594460; doi:10.1186/s12875-020-01291-x)
Supplement: Supplementary file 1 — Additional file 1. Deriving a brief measure of treatment burden to assess person-centered healthcare quality in primary care. [file 12875_2020_1291_MOESM1_ESM.pdf]

Additional File 1: Deriving a brief measure of treatment burden to assess person-centered healthcare quality in primary care

This file provides the methods and findings of the derivation phase of the study (steps 1 and 2).

Step 1: Winnowing the full 60-item PETS

In Step 1, patients with multiple chronic conditions (MCCs) and primary care providers helped winnow items from the full 60-item PETS to a subset of those most relevant to a person-centered assessment of quality in primary care. Patients with MCCs and healthcare providers who care for patients with MCCs were recruited from primary-care clinics of the Hennepin County Medical Center (HCMC) in Minneapolis, Minnesota. HCMC is Minnesota's largest 'safety net' hospital, providing care for many low-income, uninsured, and vulnerable persons living in urban Minneapolis and surrounding areas. Eligible patients were at least 21 years old, could comprehend English, had ≥ 2 chronic medical conditions (medical record confirmed), and were medical clinic outpatients with regularly scheduled appointments with a clinic provider (e.g., every 6 months). Patients with severe cognitive impairments (e.g., dementia) or other conditions that might make it difficult to complete a survey (e.g., psychoses) were excluded. Healthcare providers (any type) were eligible if they had at least 3 years' experience treating or caring for patients with MCCs.

Patient interviews: Eligible patients were identified from the medical record and clinic scheduling database and recruited in clinic by a study coordinator (MW, MV, or SP). While the sample was one of convenience, study coordinators were instructed to strive for balance in the number of males versus females and achieve a spread in the number of chronic conditions as this can be a proxy for the complexity of treatment and self-management. Thirty semi-structured interviews were conducted by a research assistant from October – December, 2016. During the

interview each patient first completed the full PETS (60-item) measure to become familiar with the items. Afterward, the patient was given a checklist featuring all PETS items and instructed to check the 20 items felt to be the "most important issues or concerns about self-management and healthcare that a healthcare provider should know about." The patient was then asked to review their 20 nominated items and select up to 10 items considered to be the "very most important." Three versions of the checklist were created, differing by item presentation order with patients randomly assigned to a version to control for order effects. Demographic data, health, and visit information were also collected. Patients were compensated $10 for the interview.

Provider surveys: Eligible healthcare providers at HCMC were identified by two study co-investigators (ML and DB) and recruited by phone by a study coordinator in October and November of 2016. Providers were purposively sampled to represent a range of provider types (e.g., physicians, nurses, pharmacists, psychologists) and years of experience caring for patients with MCCs. Thirty providers completed the winnowing exercise by survey. They first reviewed the full 60-item PETS measure and were then provided with the checklist of PETS items. Providers were instructed to check the 20 items felt to be the "most important issues or concerns about self-management and healthcare that they should know about when caring for a patient with MCCs." Of the 20 concerns nominated as "most important," providers were asked to select up to 10 considered to be "very most important." Like the patients, the providers were randomly assigned to one of three versions of the checklist to control for item ordering. Descriptive data were also collected from the survey. Each provider was compensated $25 for returning a completed survey. Both the patient interviews and provider surveys used to winnow PETS items were approved by the Hennepin Healthcare IRB under HSR #16-4200. The study was deemed minimal risk by the IRB and signed consent documentation was waived.

Findings of Step 1 winnowing: Demographic and medical characteristics of the 30 patients interviewed appear in Supplementary Table 1. Briefly, mean age of the sample was 57.5 years, 53% were male, 57% were African-American, and the median number of self-reported chronic conditions was 6.0. The types of chronic conditions reported were diverse with those most frequently reported being hypertension (77%), depression or anxiety (70%), diabetes or high blood sugar (57%) and arthritis (57%). Over half (60%) reported 6 or more visits to the primary care clinic in the past year. Descriptive statistics of the 30 healthcare providers who responded to the survey appear in Supplementary Table 2. Briefly, mean age of the provider sample was 44 years, 80% were female, and most were either physicians (40%) or nurses, including advanced nurse practitioners (30%). Half of the providers had over 10 years of experience caring for patients with MCCs.

Results of the item endorsement exercise for both patients and providers appear in Supplementary Table 3. The table contains the frequencies that each PETS item is endorsed as "very important" by the 30 patients and 30 providers separately. The frequency of endorsing an item by chance was calculated by dividing 10 (the number of allowable "very most important" items) by the total number of PETS items reviewed (60), and multiplying this by the number of participants in each exercise (30). This serves as a cutoff threshold for retaining items for the draft Brief PETS measure. As shown in the table, 13 items exceeded chance endorsement in both the patient interview and provider survey samples. These items were automatically retained for the draft Brief PETS measure. Another 14 items exceeded chance endorsement in either the patient or provider sample, but not both. These items were retained for further review and scrutiny in Step 2 of the derivation process.

Step 2: Panel review of Step 1 results and supplementary data

Results of the Step 1 item endorsement exercise as well as supplementary data on the PETS were reviewed by a panel of six co-investigators of the project. The panel consisted of two PhD-level nursing researchers (MF and CV), two physicians (ML and ER), one advanced nurse practitioner (DB), and one PhD-level measurement expert (RA). All are experienced health-services researchers. Panel members first reviewed the findings of the winnowing exercise including the patient and provider item endorsement frequencies as well as frequency distributions and descriptive statistics (e.g., means, medians) on all PETS items from the 30 patients who were administered the measure. Additionally, panel members also had access to available supplementary data from the original PETS validation study, which included responses from 332 patients with MCCs [1]. This included frequency distributions and descriptive statistics (e.g., means, medians) on all PETS items as well as correlations of each PETS item score with scores on study measures used for convergent validation (i.e., chronic condition distress, medication convenience, medication adherence, self-efficacy, and convenience of healthcare services). This supplementary item-level data from the original validation study was made available to assist panel members in resolving discordant results between patients and providers on the winnowing exercise and to consider for inclusion any other PETS items frequently endorsed by patients as problematic issues in the prior analysis (see rules for inclusion below). Clinical relevance of these items was indicated by their correlation with the study measures used for convergent validation. Panel members independently reviewed all of this item-level PETS data prior to an in-person meeting of the full panel held in early May of 2017.

During the in-person meeting all PETS items were reviewed, domain by domain, and the panel made decisions about which items to include in the draft measure. The following rules

were developed a priori and applied to determine the items to include: (Rule 1) Items exceeding chance endorsement in both patients and providers were automatically selected for inclusion. (Rule 2) Items exceeding chance endorsement in either patients or providers (but not both) were discussed by the panel followed by a vote to include. An item was included when more than 50% of panel members voted to include it. (Rule 3) Among the remaining items, supplementary item-level data were displayed for all panel members using Microsoft Excel spreadsheets. Upon review of all available data, a vote was taken with any item exceeding 50% endorsement by the panel included.

Findings of Step 2 panel review: Overall, 34 items of the original 60-item PETS measure were selected for inclusion in the draft Brief PETS measure. These items appear in bold and are highlighted in color (based on the inclusion rule met) in Supplementary Table 3. Of the items selected for inclusion, 13 met the conditions of Rule 1, 11 met the conditions of Rule 2, and 10 met the conditions of Rule 3. Regarding the inclusions under Rule 3, the panel chose to include these items based on a thorough review of the supplementary data provided. All of these items were reported to be highly frequent concerns for patients in both of the PETS administrations: the 30 patient interviews of Step 1 and the archived data from the Eton et al. validation study. The included items in the role/social limitations and physical/mental exhaustion domains were all found to be moderately-to-highly correlated with other study validation measures, particularly chronic condition distress.

References

1. Eton DT, Yost KJ, Lai JS, Ridgeway JL, Egginton JS, Rosedahl JK, Linzer M, Boehm DH, Thakur A, Poplau S *et al*: **Development and validation of the Patient Experience with Treatment and Self-management (PETS): a patient-reported measure of treatment burden**. *Qual Life Res* 2017, **26**:489-503.

Supplementary data tables (see Additional File 2)

Supplementary Table 1. Patient demographic and medical characteristics (N=30)

Supplementary Table 2. Healthcare provider descriptive characteristics (N=30)

Supplementary Table 3. PETS item endorsement frequencies of “very important” for patients and healthcare providers and items selected for the draft Brief PETS
