# Supplementary material for: Deriving and validating a brief measure of treatment burden to assess person-centered healthcare quality in primary care: a multi-method study
Source: BMC Fam Pract. 2020 Oct 28;21:221. doi: 10.1186/s12875-020-01291-x (PMC7594460; doi:10.1186/s12875-020-01291-x)
Supplement: Supplementary file 2 — Additional file 2. Supplementary data tables for derivation phase. [file 12875_2020_1291_MOESM2_ESM.pdf]

Additional File 2: Supplementary data tables for derivation phase

Supplementary Table 1. Patient demographic and medical characteristics (N=30) [derivation phase]

**Mean age in years (SD)**	57.5 (9.7)
Range	38 – 83
Median	57
**Gender (N, %)**	
Male	16 (53%)
Female	14 (47%)
**Race (N, %)**	
Black / African-American	17 (57%)
White	12 (40%)
Asian	1 (3%)
**Marital status (N, %)**	
Separated, divorced, or widowed	17 (57%)
Never married	12 (40%)
Married or living with a partner	1 (3%)
**Education level (N, %)**	
Less than High school	6 (20%)
High school graduate	6 (20%)
Some college / Associate's degree	13 (43%)
College graduate (B.A., B.S.)	4 (13%)
Graduate school / Advanced degree	1 (3%)
**Work status (N, %)**	
On disability or leave	16 (53%)
Retired or unemployed	9 (30%)
Working full or part-time	4 (13%)
Full-time student	1 (3%)
**Yearly income (N, %)**	
< $20,000	26 (87%)
$20,000 - $30,000	2 (7%)
$30,000 - $60,000	0 (0%)
$60,000 - $80,000	1 (3%)
> $80,000	0 (0%)
Missing	1 (3%)
**Living situation (N, %)**	
Living in apartment or home	22 (73%)
Homeless	5 (17%)
Other (room and board)	2 (7%)
Assisted living or nursing home	1 (3%)
**No. people residing in home or apartment (N, %)**	
1	17 (57%)
2-3	4 (13%)
4+	2 (7%)

Missing	7 (23%)
**Self-reported conditions (N, %)**	
Hypertension	23 (77%)
Depression or anxiety	21 (70%)
Diabetes or high blood sugar	17 (57%)
Arthritis	17 (57%)
Emphysema/asthma/bronchitis	15 (50%)
Chronic backache	13 (43%)
Vision problems (e.g., glaucoma, macular degeneration, cataracts)	11 (37%)
Obesity	11 (37%)
Other*	10 (33%)
Substance abuse disorder	7 (23%)
Osteoporosis	7 (23%)
Hepatitis	7 (23%)
Angina or heart-related chest pain	6 (20%)
Atrial fibrillation or other heart arrhythmia	5 (17%)
Chronic atopic dermatitis or psoriasis	5 (17%)
Migraine headache	5 (17%)
Hearing problems	5 (17%)
Myocardial infarction or heart attack	3 (10%)
Heart failure	3 (10%)
Coronary artery disease	3 (10%)
Stroke or brain hemorrhage	3 (10%)
Chronic kidney disease	3 (10%)
Cancer	2 (7%)
HIV	2 (7%)
Colitis, Crohn's Disease, or Irritable bowel syndrome	1 (3%)
*Other: Bipolar, Chronic obstructive pulmonary disease, Graves Disease, Hip replacement complications, Foot problems, Nerve pain, Posttraumatic stress disorder, Rotator cuff injury, Sleep apnea, Traumatic brain injury	
**No. of self-reported conditions (N, %)**	
2-4	7 (23%)
5-6	9 (30%)
7-8	7 (23%)
9+	7 (23%)
Median	6.0
**No. of visits to primary medicine clinic in past year (N, %)**	
1	1 (3%)
2-3	3 (10%)
4-5	7 (23%)
6-7	3 (10%)
8+	15 (50%)
Missing	1 (3%)

Supplementary Table 2. Healthcare provider descriptive characteristics (N=30) [derivation phase]

**Mean age in years (SD)**	44.0 (12.3)
Range	30 – 66
Median	39
**Gender (N, %)**	
Female	24 (80%)
Male	4 (13%)
Missing	2 (7%)
**Provider type (N, %)**	
Physician	12 (40%)
Nurse (incl. advanced practice nurse practitioner)	9 (30%)
Pharmacist	2 (7%)
Psychologist	2 (7%)
Community health worker	2 (7%)
Other*	2 (7%)
Missing	1 (3%)
*Other: Certified diabetes educator (1), dietician (1)	
**Department (N, %)**	
General medicine clinic	22 (73%)
Pharmacy	2 (7%)
Psychiatry	2 (7%)
Nutrition services	1 (3%)
Case management	1 (3%)
Missing	2 (7%)
**Years of experience caring for MCC patients (N, %)**	
Over 20	9 (30%)
16-20	0 (0%)
11-15	6 (20%)
6-10	7 (23%)
3-5	7 (23%)
Missing	1 (3%)
**No. of patients with MCC ever treated/managed by provider (N, %)**	
2000 or more	13 (43%)
1000-1999	4 (13%)
500-999	8 (27%)
100-499	4 (13%)
Missing	1 (3%)
**Full- or Part-time work status (N, %)**	
Full-time	18 (60%)
Part-time	11 (37%)
Missing	1 (3%)

MCC: Multiple chronic conditions

Supplementary Table 3. PETS item endorsement frequencies of "very important" for patients and healthcare providers and items selected for the draft Brief PETS[a] [derivation phase]

**Medical information items**	**Endorsement frequency by patients (N=30)**	**Endorsement frequency by providers (N=30)**	**Exceeding chance[b] in both patients AND providers**	**Exceeding chance[b] in either patients OR providers**
**Learn about your health problem(s)?**	7 (23%)	10 (33%)	√	
**Learn what foods you should eat to stay healthy?**	7 (23%)	1 (3%)		X
Find information on the medications that you have to take?	5 (17%)	0 (0%)		
**Understand any changes to your treatment plan?**	6 (20%)	8 (27%)	√	
**Understand the reasons why you are taking some medicines?**	7 (23%)	10 (33%)	√	
**Find sources of medical information that you trust?**	5 (17%)	2 (7%)		
**Understand advice from different healthcare providers?**	6 (20%)	8 (27%)	√	
**Medication items**	**Endorsement frequency by patients (N=30)**	**Endorsement frequency by providers (N=30)**	**Exceeding chance[b] in both patients AND providers**	**Exceeding chance[b] in either patients OR providers**
Organize your medicines?	4 (13%)	4 (13%)		
**Take more than one medicine every day?**	8 (27%)	1 (3%)		X
Take your medicines several times each day?	2 (7%)	7 (23%)		X
Refill your medicines?	2 (7%)	4 (13%)		
Adjust your medicines (including the amount, type, or time when you take it)?	3 (10%)	1 (3%)		
**Take your medicines as directed?**	4 (13%)	13 (43%)		X
Plan your daily activities around your medicine schedule?	2 (7%)	1 (3%)		
**Medication bother items**	**Endorsement frequency by patients (N=30)**	**Endorsement frequency by providers (N=30)**	**Exceeding chance[b] in both patients AND providers**	**Exceeding chance[b] in either patients OR providers**
How much you have to rely on your medicine(s)?	4 (13%)	0 (0%)		
**Side effects of your medicine(s)?**	11 (37%)	4 (13%)		X

**Medical appointment items**	**Endorsement frequency by patients (N=30)**	**Endorsement frequency by providers (N=30)**	**Exceeding chance[b] in both patients AND providers**	**Exceeding chance[b] in either patients OR providers**
**Make or keep your medical appointments?**	3 (10%)	13 (43%)		X
Schedule and keep track of your medical appointments?	2 (7%)	6 (20%)		X
Make or keep appointments with different healthcare providers?	5 (17%)	0 (0%)		
Find the time to get to your medical appointments?	1 (3%)	2 (7%)		
Find the energy to get to your medical appointments?	2 (7%)	0 (0%)		
**Find transportation to get to your medical appointments?**	10 (33%)	13 (43%)	√	
**Monitoring health items**	**Endorsement frequency by patients (N=30)**	**Endorsement frequency by providers (N=30)**	**Exceeding chance[b] in both patients AND providers**	**Exceeding chance[b] in either patients OR providers**
**Monitor your health behaviors, for example, tracking your exercise, the foods you eat, or medicines you take?**	6 (20%)	9 (30%)	√	
**Monitor your health condition, for example, weighing yourself, checking your blood pressure, or checking your blood sugar?**	6 (20%)	9 (30%)	√	
**Diet items**	**Endorsement frequency by patients (N=30)**	**Endorsement frequency by providers (N=30)**	**Exceeding chance[b] in both patients AND providers**	**Exceeding chance[b] in either patients OR providers**
**I have to give up too many foods that I like.**	4 (13%)	0 (0%)		
It is hard to find healthy foods.	5 (17%)	2 (7%)		
**It is hard for me to follow my healthcare provider's recommendations for healthy eating.**	6 (20%)	8 (27%)	√	
**Exercise or physical therapy items**	**Endorsement frequency by patients (N=30)**	**Endorsement frequency by providers (N=30)**	**Exceeding chance[b] in both patients AND providers**	**Exceeding chance[b] in either patients OR providers**
It is difficult for me to find the time to exercise or do physical therapy	3 (10%)	3 (10%)		
It is difficult for me to follow my healthcare provider's recommendations about exercise or physical therapy	1 (3%)	7 (23%)		X
**It is difficult for me to get motivated to exercise or do physical therapy**	7 (23%)	4 (13%)		X

**Physical pain or discomfort limits my ability to exercise or do physical therapy**	9 (30%)	3 (10%)		X
**Medical equipment items**	**Endorsement frequency by patients (N=30)**	**Endorsement frequency by providers (N=30)**	**Exceeding chance[b] in both patients AND providers**	**Exceeding chance[b] in either patients OR providers**
Use your medical equipment or device?	0 (0%)	4 (13%)		
Keep your medical equipment or device working correctly?	1 (3%)	1 (3%)		
**Relationships with others items**	**Endorsement frequency by patients (N=30)**	**Endorsement frequency by providers (N=30)**	**Exceeding chance[b] in both patients AND providers**	**Exceeding chance[b] in either patients OR providers**
Feeling dependent on others for your healthcare needs?	2 (7%)	1 (3%)		
Others reminding you to do things for your health like take your medicine, watch what you eat, or schedule medical appointments?	3 (10%)	0 (0%)		
**Your healthcare needs creating tension in your relationships with others?**	6 (20%)	2 (7%)		X
**Others not understanding your health situation?**	6 (20%)	4 (13%)		X
**Medical and healthcare expenses items**	**Endorsement frequency by patients (N=30)**	**Endorsement frequency by providers (N=30)**	**Exceeding chance[b] in both patients AND providers**	**Exceeding chance[b] in either patients OR providers**
Plan for the future because of your medical expenses?	2 (7%)	2 (7%)		
**Pay for healthy foods?**	11 (37%)	7 (23%)	√	
**Pay for all of your medical expenses?**	6 (20%)	11 (37%)	√	
**Pay for your medicines?**	6 (20%)	13 (43%)	√	
**Understand what is and what is not covered by your health insurance?**	6 (20%)	3 (10%)		X
**Difficulty with healthcare services items**	**Endorsement frequency by patients (N=30)**	**Endorsement frequency by providers (N=30)**	**Exceeding chance[b] in both patients AND providers**	**Exceeding chance[b] in either patients OR providers**
**I have problems with different healthcare providers not communicating with each other about my medical care**	2 (7%)	5 (17%)		
**I have to see too many different specialists for my health problem(s) or illness(es)**	4 (13%)	5 (17%)		

I have problems filling out forms related to my healthcare	4 (13%)	3 (10%)		
I have problems getting appointments at times that are convenient for me	1 (3%)	4 (13%)		
I have problems getting appointments with a specialist	2 (7%)	0 (0%)		
I have to wait too long at my medical appointments	3 (10%)	4 (13%)		
I have to wait too long at the pharmacy for my medicine	2 (7%)	1 (3%)		
**Role / social activity limits due to self-management items**	**Endorsement frequency by patients (N=30)**	**Endorsement frequency by providers (N=30)**	**Exceeding chance[b] in both patients AND providers**	**Exceeding chance[b] in either patients OR providers**
**Work (include work at home)?**	3 (10%)	4 (13%)		
Family responsibilities?	1 (3%)	2 (7%)		
**Daily activities?**	2 (7%)	14 (47%)		X
**Hobbies and leisure activities?**	2 (7%)	2 (7%)		
Ability to spend time with family and friends?	0 (0%)	3 (10%)		
**Ability to travel for work or vacation?**	5 (17%)	2 (7%)		
**Physical / mental exhaustion due to self-management items**	**Endorsement frequency by patients (N=30)**	**Endorsement frequency by providers (N=30)**	**Exceeding chance[b] in both patients AND providers**	**Exceeding chance[b] in either patients OR providers**
**Angry?**	3 (10%)	1 (3%)		
**Preoccupied?**	2 (7%)	0 (0%)		
**Depressed?**	11 (37%)	10 (33%)	√	
**Worn out?**	5 (17%)	1 (3%)		
**Frustrated?**	11 (37%)	7 (23%)	√	

Notes: [a]Items in **bold** and highlighted in color were those selected for inclusion in the Brief PETS measure to be tested in the validation study of Step 3. Yellow highlighting indicates the item met the Rule 1 inclusion criterion. Blue highlighting indicates the item met the Rule 2 inclusion criterion. Green highlighting indicates the item met the Rule 3 inclusion criterion. [b]The cut-off for chance endorsement is 5 (17%); Chance endorsement = 10 (number of allowable "very important" items) / 60 (total number of PETS items judged) x 30 (number of participants judging). Endorsement ≥ 6 or at least 20% of participants exceeds chance.
