# Supplementary material for: Deriving and validating a brief measure of treatment burden to assess person-centered healthcare quality in primary care: a multi-method study
Source: BMC Fam Pract. 2020 Oct 28;21:221. doi: 10.1186/s12875-020-01291-x (PMC7594460; doi:10.1186/s12875-020-01291-x)
Supplement: Supplementary file 3 — Additional file 3: Appendix: The Brief Patient Experience with Treatment and Self-management (Brief PETS). [file 12875_2020_1291_MOESM3_ESM.pdf]

Appendix. The Brief Patient Experience with Treatment and Self-management (Brief PETS) version 1.0[a]

DOMAIN / ITEMS
[b]***Medical information:*** How easy/difficult has it been to …<br>(Responses: very easy, easy, neither easy nor difficult, difficult, very difficult, not applicable)
learn about your health problem(s)?
learn what foods you should eat to stay healthy?
understand changes to your treatment plan?
understand the reasons why you are taking some medicines?
find sources of medical information that you trust?
understand advice from different healthcare providers?
[b]***Medications:*** How easy/difficult has it been to …<br>(Responses: very easy, easy, neither easy nor difficult, difficult, very difficult)
take more than one medicine every day?
take your medicines as directed?
[b]***Medical appointments:*** How easy/difficult has it been to …<br>(Responses: very easy, easy, neither easy nor difficult, difficult, very difficult)
make or keep your medical appointments?
find transportation to get you to your medical appointments?
[b]***Monitoring health:*** How easy/difficult has it been to …<br>(Responses: very easy, easy, neither easy nor difficult, difficult, very difficult, not applicable)
monitor your health behaviors, e.g., tracking exercise, foods you eat, or medicines you take?
monitor your health condition, e.g., weighing yourself, checking blood pressure, or checking blood sugar?
Medication side-effects bother: How bothered have you been by ...<br>(Responses: not at all, a little, somewhat, quite a bit, very much)
side effects of your medicine(s)?
Diet: Has a doctor, nurse, dietician or other healthcare provider discussed or given you recommendations for healthy eating (Yes/No)?<br>If *Yes*, then: How much do you agree or disagree with the following statements?<br>(Responses: strongly agree, agree, disagree, strongly disagree)
I have to give up too many foods that I like.
It is hard for me to follow my healthcare provider's recommendations for healthy eating.
Exercise or physical therapy: Has a doctor, nurse, physical therapist or other healthcare provider discussed or recommended exercise or physical therapy specifically for your health problem(s) or illness(es) (Yes/No)?<br>If *Yes*, then: How much do you agree or disagree with the following statements?<br>(Responses: strongly agree, agree, disagree, strongly disagree)
It is difficult for me to get motivated to exercise or do physical therapy.
Physical pain or discomfort limits my ability to exercise or do physical therapy.

Medical & healthcare expenses: How easy/difficult has it been for you to … (Responses: very easy, easy, neither easy nor difficult, difficult, very difficult, not applicable)
pay for healthy foods?
pay for all of your medical expenses?
pay for your medicines?
understand what is and what is not covered by your health insurance?
Difficulty with healthcare services: How much do you agree/disagree with the following? (Responses: strongly agree, agree, disagree, strongly disagree, not applicable)
Have problems with different healthcare providers not communicating with each other about my medical care
Have to see too many different specialists for my health problem(s) or illness(es)
[c]***Role and social activity limitations:*** How much has your self-management interfered with … (Responses: not at all, a little, somewhat, quite a bit, very much)
work (include work at home)?
daily activities?
hobbies and leisure activities?
ability to travel for work or vacation?
[c]***Physical and mental exhaustion:*** How often did your self-management make you feel … (Responses: never, rarely, sometimes, often, always)
angry?
preoccupied?
depressed?
worn out?
frustrated?

Notes. [a]Brief PETS Version 1.0, © 2020 Mayo Foundation for Medical Education and Research, reprinted with permission. All right reserved. [b]Domains that make up the "workload" summary index score. [c]Domains that make up the "impact" summary index score. Self-management is defined for respondents as "all of those tasks and activities that you have to do specifically for your health problem(s) or illness(es) in order to stay healthy." With the exception of items in the "Difficulty with healthcare services" domain, all items reference a recall time period of the *past 4 weeks*. No recall time period is used for the difficulty with healthcare services items.
